# Assessing the accessibility and quality of mobile health applications for the treatment of obesity in the German healthcare market

**DOI:** 10.3389/frhs.2024.1393714

**Published:** 2024-06-11

**Authors:** Pia M. Stapelfeldt, Sina A. M. Müller, Linda Kerkemeyer

**Affiliations:** LiKe Healthcare Research GmbH, Berlin, Germany

**Keywords:** smartphone, mobile apps, mobile health, obesity, weight management, behavior change techniques

## Abstract

**Introduction:**

Overweight and obesity are among the most prevalent health problems worldwide leading to various diseases and having a significant impact on the healthcare system. In Germany, the prevalence of obesity among adults is 19%. Mobile health applications offer a new approach to treatment and prevention and have been proven effective in previous studies. However, it is essential to investigate the availability and quality of these digital applications. The aim of this systematic assessment is to evaluate the accessibility and quality of digital health applications in German language designed to treat obesity.

**Methods:**

In January 2024, a systematic search for mobile health applications was conducted on both the Google Play Store and Apple App Store. Just those apps available in German for both iOS and Android were considered acceptable. The German Mobile Application Rating Scale (MARS-G) was used to assess the quality of the apps. The content of mobile health applications was evaluated using the guideline from the German Obesity Society for the treatment of obesity. The characteristics of the apps were summarized and presented, and the results were analyzed using descriptive statistics and presented in tables.

**Results:**

After screening, ten apps were included in the review. The apps varied in terms of calorie tracking, individual workout plans, educational aspects, nutritional plans, and exercises for behavioral change. On average, 6.4 out of 12 items of the German Obesity guideline recommendations were fulfilled. The MARS score (possible range from 1–5) reached a mean of 3.39 (SD = 0.39). The section “Engagement” had the lowest quality score with a mean of 3.14 (SD = 0.57), while the section “Aesthetics” achieved the highest mean of 3.57 (SD = 0.52).

**Discussion:**

Most German mobile health applications for managing obesity meet some guideline recommendations. They demonstrate adequate to good quality according to the MARS score. Assessing the quality of mobile health applications can be challenging for patients, despite being easily accessible and low-threshold. However, such digital health applications, reimbursed by the German SHI, offer evidence-based information, even if access can be associated with higher hurdles.

## 1 Introduction

The World Health Organization (WHO) defines obesity in the ICD-11 (International Classification of Diseases 11th revision) as a chronic, complex disease caused by a variety of factors, such as psychosocial or genetic causes. Obesity is mostly measured by the body mass index (BMI), which is calculated from height and weight. A BMI of ≥30.0 kg/m^2^ is classified as obesity and can be further categorized as class I (30.0–34.9 kg/m^2^), class II (35.0–39.9 kg/m^2^), or class III (≥40.0 kg/m^2^) to determine appropriate treatment options (1).

Based on data from the Global Burden of Disease (GBD) study, obesity had a global prevalence of 14% in 2019 (2). Furthermore, approximately five million deaths in 2019 were attributed to obesity (3). This indicates that obesity is a growing global problem, affecting healthcare systems in different countries (4). The GBD study also assumed that the number of Disability-adjusted Life Years (DALYs) due to obesity will increase by 3.4% annually worldwide by 2030, resulting in an overall increase in obesity-related DALYs of 39.8% within one decade (3).

Germany is no exception in these developments regarding obesity. Based on a national, representative study using self-completed questionnaires of the German population to collect information on body height and weight, a total of 53.5% of German adults were overweight or obese in 2019 and 2020 (5). Over the last ten years, the prevalence of obesity has increased. Based on this study from 2019–2020, 13 million German adults, equivalent to 19% of adults in Germany, were affected by obesity (5).

The increasing number of patients affected by obesity also amplifies the economic burden. An international study has analyzed the economic impact of overweight and obesity in 161 countries based on direct and indirect costs from a societal perspective and calculated a loss in global Gross Domestic Product (GDP) of around 2.19% in 2019 (6). In addition, the loss of GDP due to the effects of obesity and overweight is estimated at 3.29% globally by 2060 (6).

In Germany, obesity-related medical costs increased by 193 million euros in five years, leading to more than one billion euros spent on obesity in 2020 (7). A study examining the costs associated with obesity in Germany, using a prevalence and life-cycle perspective, found that healthcare costs per quarter increase with BMI class. For BMI class I, there are quarterly additional costs of € 314.96 per patient, rising to € 631.64 for BMI class III (8).

Obesity is associated with several non-communicable diseases and comorbidities. Therefore, obese people have a higher risk of developing diabetes mellitus, hypertension, coronary heart diseases, a stroke, Alzheimer's disease, or certain types of cancer (9, 10), resulting in various diseases in different organ systems associated with obesity. People affected by obesity are five times more likely to develop a simple multimorbidity and 12 times more likely to develop a complex multimorbidity (10).

In addition to the increased risk of multimorbidity, affected individuals must also cope with various psychosocial impacts. While mental illness is a risk factor for developing obesity (11), there is a reciprocal link found for depression and obesity (12). Various adverse interacting aspects are discussed, such as possible metabolic changes due to medication, reduced exercise due to a lack of drive (11, 13), but also emotional eating (13, 14). Regardless of the development of pathologies, obese people often experience stigmatization in different parts of their lives. These issues include marginalization, teasing and prejudice in society and the healthcare sector, leading to professional disadvantages (13, 15). Thus, our society is centered on the average individual, people with obesity must deal with various difficulties, including small chairs in public spaces, narrow changing rooms, and limited clothing options (16). All these aspects result in psychological strain. This has to be taken into account when choosing a treatment and, if necessary, combine it with additional psychotherapy (17).

Several guidelines for effective preventive measures and evidence-based, optimal treatment of obesity have been developed as recommendations for healthcare professionals and patients. They are intended to support decision-making on the adequate treatment of obesity. Both the German Obesity Society guideline *(Deutsche Gesellschaft für Adipositas)* and the National Institute for Health and Care Excellence (NICE) guideline recommend several approaches for a successful management of the disease (13, 18). These include dietary therapy, exercise therapy, behavioral therapy (13, 18), and lifestyle interventions (18). Besides treatment options, the German guideline specifies various preventive actions, which mostly concentrate on dietary recommendations, as well as suggestions for sufficient physical activity (13). The guideline from the German Obesity Society also mentions the potential use of weight loss programs, mostly containing self-management aspects that should fulfill quality requirements (13).

In April 2024, a disease management program (DMP) for the treatment of obesity in Germany came into force. A DMP is a special structured treatment program for selected chronic diseases which is based on the findings of evidence-based medicine and involves expertise from General Practitioners (GPs) as well as specialists. The obesity DMP will probably consist of multimodal training, focusing on individual fitness and nutritional recommendations as well as suggestions for behavioral changes (19). Access is given to patients by enrolling in a DMP at their statutory health insurance (SHI) company, coordinated by their general practitioner (GP) (20).

In the era of digitalization, more and more programs for the most important lifestyle changes such as smoking cessation, healthy diet, weight reduction, and adherence to the regular practice of physical exercises are being offered and used as mobile health applications (21, 22, 23). Smartphones and wearables, such as smartwatches or activity tracker, can be used to measure and record vital parameters and other health-related data using a variety of sensors. Interested users can download the mobile health applications from the app store, either free of charge or as a self-payment option. These apps, which are mostly used for a self-management, are accessible to anyone with a mobile device.

The Digital Healthcare Act *(Digitale-Versorgungs-Gesetz* or *DVG),* which came into force in 2020, represents a change in the German healthcare system. Since then, physicians have the option of prescribing digital health applications (*Digitale Gesundheitsanwendungen* or *DiGA*) for the treatment of various diseases (24). A DiGA is a medical device whose main functions are based on digital technologies. Thereby, the DiGA is only used by the patient or together by the patient and the healthcare professional. In contrast to mobile health applications downloaded for free from the app store, the DiGA is not solely a digital application used for data collection from a device or for device control. Instead, the DiGA is aimed at recognizing, monitoring, treating or alleviating diseases, injuries, or disabilities. Insured persons of the SHI have the possibility to receive a DiGA, which is financed by their health insurance. Therefore, the DiGA must demonstrate a positive care effect, either as a medical benefit or an improvement of the procedure, by conducting a comparative study. If the DiGA can demonstrate a positive proof of effectiveness, the DiGA will be permanently included in the DiGA directory. It is also possible to become a provisional listed DiGA for one year, even if the evidence has not yet been proven. In this case, an ongoing study is carried out, to demonstrate a positive care effect during the one-year period (25). Currently 57 DiGA are listed or provisional listed (status: 23 May 2024) in the DiGA directory (26). The listed DiGA address various diseases, also showing two digital health applications for the treatment of obesity: “Oviva Direkt” and “zanadio” (26, 27).

The aim of this review is to identify mobile health applications for self-treatment of obesity. The mobile health applications will be evaluated in terms of content, quality, and accessibility. In particular, the use of the mobile health apps from the perspective of the patients is essential.

## 2 Methods

### 2.1 Search and screening

A systematic search for smartphone apps for Apple iOS and Google Android was carried out. For this purpose, the Apple App Store was searched for iOS apps and the Google Play Store was searched for Android apps. The final search was carried out in January 2024. Relevant German synonyms for obesity and overweight were used as search terms. Each term was searched individually in the two app stores by one reviewer (PMS). The following search terms were used: “*Adipositas”* (obesity), “*Übergewicht”* [overweight (as a noun)], “*übergewichtig”* [overweight (as an adjective)], *“adipös”* (obese), “*Gewichtsreduktion”* (weight reduction)*, “Gewichtsverlust”* (weight loss), “*abnehmen”* (lose weight), “*Gewichtsabnahme”* (weight loss), “*Diät”* (diet). No other search filters were used.

The relevant app data were collected in a table by this reviewer (PMS). This included the app name, age recommendation, developer, the latest update, the average rating in the store, the number of ratings given, and the description associated with the app. Any duplicates found during the search were excluded.

Afterwards, the screening process began. The first step was to check the list to see whether the apps were available in both the Apple App Store and the Google Play Store. If not, the app was excluded. In the next step, the names of the apps and the descriptions from the App Store were screened and examined for the inclusion criteria. This step is like the abstract screening in a systematic review, in which non-matching hits are excluded. The previously defined inclusion criteria related to the app being in German, being available free of charge (for at least 14 days as a trial version), being available in both the Apple App Store and Google Play Store and functioning independently (no need for external additional devices or a membership). These criteria aim to make the apps easily accessible and ensure that all patients—regardless of their smartphone—can use them. The app should also cover at least two of the following topics according to the guideline: nutritional therapy, diet programs, exercise therapy or behavioral therapy. As a final screening step, the remaining apps were downloaded, tested, and examined for at least ten minutes by two reviewers (PMS and LK). After the examination, these apps were checked again regarding the inclusion criteria. The two reviewers (PMS and LK) discussed uncertainties regarding the reviewed applications, and if they could not agree, the third reviewer (SAMM) was consulted to reach a consensus. The assessment was methodically adapted from similar studies (28, 29, 30).

### 2.2 Outcomes

The apps included in this review were assessed for their quality using the MARS-G, the German version of the mobile app rating scale (31, 32), and for their evidence using a checklist based on the German guideline for the prevention and treatment of obesity (13).

The MARS-G scale initially records descriptive information about the application, such as the manufacturer, name of the app or when the last update was conducted. Afterwards, the items of the MARS-G tool are divided into six quality categories. Each item can be answered using a Likert scale with five answer options (1 = inadequate to 5 = excellent). With these answers, it is possible to calculate the mean score of each category and finally the overall mean score of the app. The first section A focuses on the engagement including the entertainment, customization, interactivity, target group, and interest. After checking the first quality dimension, the second section B concentrates on the functionality of the mobile application. The subjects of performance, usability, navigation, and gestural design are highlighted. The third section C, which concentrates on the aesthetics, evaluates the layout, graphics, and visual appeal. Then, fourth section D assesses the information quality, including the accuracy of the app description presented in the store, goals, quality of information, quantity of information, visual information, credibility, and if the information is evidence-based (31, 32). The German version of the MARS tool additionally focuses on the therapeutic gain of the application (31). This additional dimension as well as the subjective quality of the app are not included in the overall mean score (31, 32). The MARS-G tool showed similar interrater reliability (ICC = 0.83) as the MARS tool (ICC = 0.84) and proved a good internal consistency (*ω* = 0.82) (28). The raters (PMS and LK) trained using the MARS-G tool according to a training video (33).

To ensure both the quality and content of mobile health applications are evaluated, the German guideline for the prevention and treatment of obesity (13) is used to assess the evidence. To check whether the content of the applications agree with the guideline, a checklist was developed. Therefore, the guideline was checked for recommendations regarding the treatment of obesity and those suggestions were formed into items which could be answered with “yes”, “no” or “unclear”. The checklist contains items based on the main topics nutritional therapy, diet programs, as well as exercise and behavioral therapy. Items contained, whether individual nutritional recommendations were given, the patient was educated about dietary changes, the patient's preferences were taken into account, diets were recommended, and different nutritional strategies were presented. It was also checked, if a step-by-step approach to weight-reduction was explained, if the user was encouraged for physical activity, and whether the physical activity is safe to execute. The last four items contained, whether the user was educated on the health benefits of physical activity apart from weight loss, the app helped to set relatable goals, a stabilization of the weight after the weight loss was encouraged, and whether behavioral therapy interventions, containing different elements, were included. These elements for example include self-monitoring of the behavior, cognitive restructuring, problem-solving training, reinforcement strategies or strategies for dealing with weight gain (13).

## 3 Results

**Figure 1:**
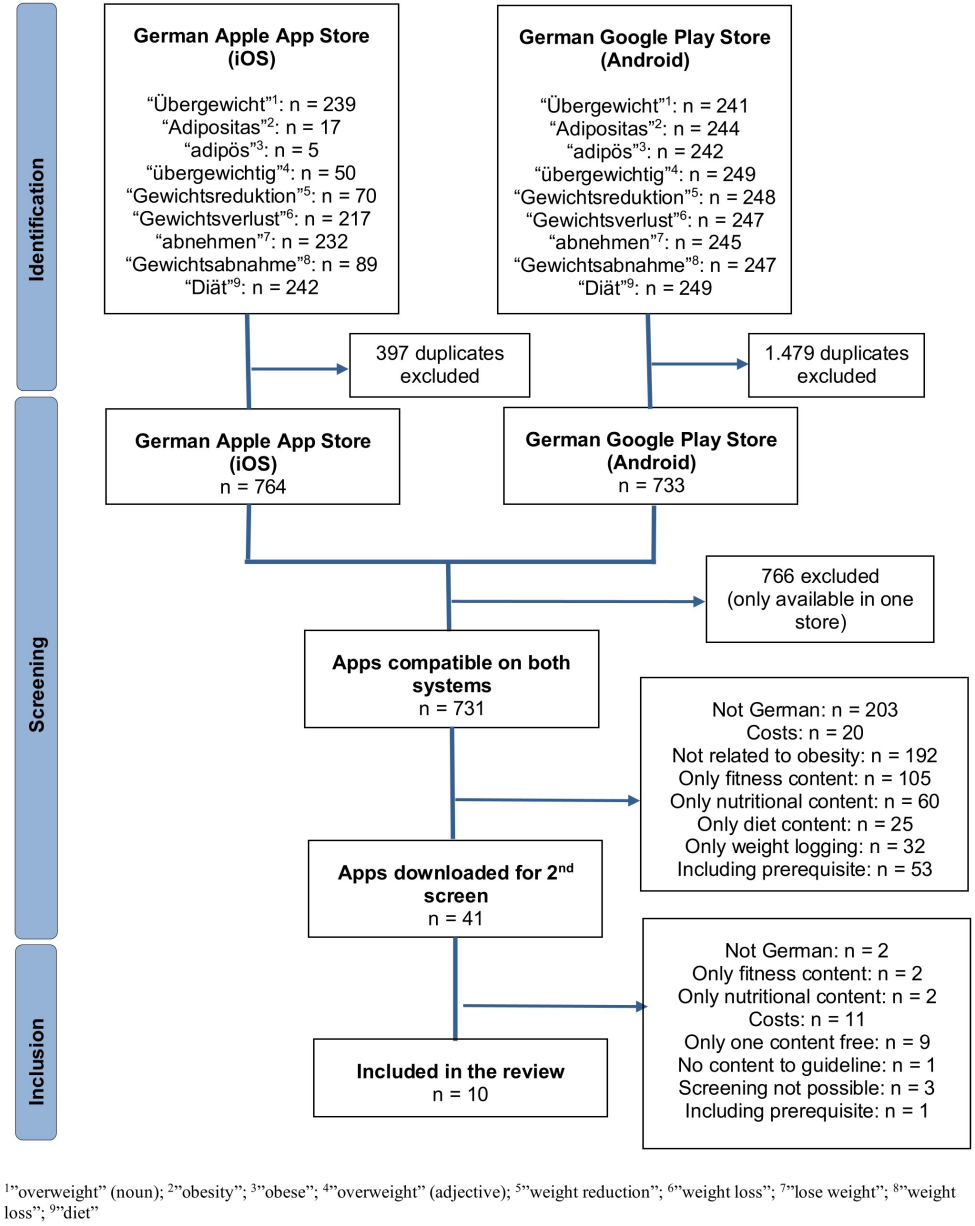
Identification process of the included apps.

In total, the search in both app stores identified 731 mobile apps, after excluding duplicates from each app store and checking if each app was available in both the Google Play Store and the Apple App Store. During the first screening process, checking the title and description of the apps, 690 applications were excluded based on the defined inclusion criteria, leaving 41 apps for the final, second screening after the apps were downloaded. Testing the apps after the download excluded 31 apps. As a result, ten mobile applications were included in the assessment, two of them are DiGA (“Oviva Direkt”, “zanadio”) and therefore the costs are reimbursed by the SHI. The identification process and details about the exclusions can be seen in a flow chart diagram based on the PRISMA statement (34) (Figure 1).

The main attributes of the ten apps were collected in Table 1, including the app name as displayed in the Apple App Store and Google Play Store, the developer, the last update, the app characteristics, the MARS-G mean score, and the content based on the German obesity guideline. The content could therefore be divided into nutritional therapy, diet programs, exercise therapy, and behavioral therapy (13). The item characteristics refer to the elements commonly found in the mobile health applications.

**Table 1:** Attributes of the assessed apps.

App name iOS (Version)	App name Android	Developer	Last update	Characteristics	MARS-G mean score	Content based on guideline
DWP Fitness—Diät & Sport (2.8)	DWP Fitness—Diät- und Sport	DWP Creation	05.01.2024	- Calorie tracking	3.15	NT, ET
				- Individual workout plan		
				- Educational content		
Fabulous—Gewohnheitstracker (1.49.1)	Fabulous—Gewohnheitstracker	Fabulous	08.01.2024	- Individual workout plan	3.78	NT, ET, BT
				- Exercises for behavioral change		
FIT-UP: Fitness & Ernährung (368)	FIT-UP: Fitness & Ernährung	Shahab Daban	31.12.2023	- Individual workout plan (live workouts)	3.15	NT, ET
				- Nutritional plan		
FizzUp: Training und Ernährung (4.6.6)	FizzUp: Training und Ernährung	FizzUp	22.01.2024	- Individual workout plan	2.93	NT, ET
				- Nutritional plan		
Foodvisor—Ernährung und Diät (6.8.0)	Foodvisor—Ernährung & Diät	Foodvisor	22.01.2024	- Calorie tracking	3.82	NT, DP, BT^a^
				- Educational content		
				- Daily courses with behavioral and educational aspects^a^		
GlücksFigur—Abnehmen & Diät (1.7.18)	GlücksFigur—Abnehmen & Diät	Lukas Mausebrink	10.01.2024	- Fitness challenges	3.0	NT, ET, BT
				- Calorie tracking		
				- Educational content		
wikifit—Kalorienzähler (1.15.5)	wikifit—Kalorienzähler	Paul Thomae	22.12.2023	- Calorie tracking	3.32	NT, ET, DP
				- Individual workout plan		
				- Diet/nutritional plan		
Workouts Zuhause—Training (19.6.3)	Spartan Home Workouts	Tech 387 LLC	01.10.2021	- Calorie tracking	3.07	NT, ET
				- Individual workout plan		
				- Nutritional plan		
Oviva Direkt (1.47.0)^b^	Oviva Direkt	Oviva AG	22.01.2024	- Nutritional plan	3.89	NT, ET, BT
				- Educational content via lections with educational and behavioral aspects		
				- Personal nutritional counseling		
				- Fitness challenges		
zanadio (1.1.46)^b^	zanadio	Sidekick Health Germany GmbH	11.01.2024	- Educational content via lections with educational, behavioral, fitness aspects	3.85	NT, ET, BT
				- Calorie tracking		
				- Challenges (fitness, nutrition, behavior)		
				- Coaching chats		

NT, nutritional therapy; DP, diet program; ET, exercise therapy; BT, behavioral therapy.

^a^ A part of the app is in English.

^b^ Official “Digitale Gesundheitsanwendungen” (DiGA) (accessed via test access).

The most common elements, included in six of ten apps, were a calorie tracking tool as well as individual workout plans. Five applications included some type of educational content, of which three also focused on behavioral changes. Some sort of nutritional plan was delivered in five apps. The inclusion of challenges (three apps), a coaching chat (one app), and the opportunity of a personal nutritional coaching (one app) were much less common in the identified apps. The apps each showed different combinations of those characteristic elements.

Looking at the latest updates for each of the included apps, nine out of ten apps have been updated within the last two months (status: 25 January 2024). Only one app was last updated in October 2021. Moreover, each of the apps included content based on nutritional therapy, and nine applications had elements from the field of exercise therapy. However, two apps addressed diet programs, and five apps contained behavioral therapy elements. In one of those apps the behavioral aspects were only accessible in English, whereas the remaining content was in German.

To evaluate whether the apps fulfilled the items listed in the German obesity guideline, each app was individually checked for including the recommended content. Overall, the mean of items answered with “yes” was higher than the mean of “no” (6.4 vs. 4.6). The mean of items answered with “unclear” was 1.0. Table 2 shows that item 7, “Encouragement of physical activity”, was mostly answered with “yes” and was found in nine out of ten applications (90%). Item 4 “Recommendations for diets” was least often answered with “yes” (10%), but three apps (30%) were classified “unclear” in this regard. The highest number of “no”'s was found in item 8 “Safety of physical activity”, which was not included in eight apps (80%). Item 1 “Individual nutritional recommendations” (70%), item 3 “Patient preferences” (80%), item 6 “Approach to weight reduction” (70%) and item 10 “Setting relatable goals” (70%) were also mostly answered with “yes”. Hence, item 11 “Encouraging weight stabilization” and item 4 “Recommendations for diets” was each answered with “no” in six times (60%). The answer “unclear” was not once given in six items: “Individual nutritional recommendations”, “Education on change of diet”, “Patient preferences”, “Approach to weight-reduction”, “Safety of physical activity”, “Behavioral therapy interventions”.

**Table 2:** Compliance of the apps with the German obesity guideline.

Item	Yes		No		Unclear	
1. Individual nutritional recommendations	7	70%	3	30%	0	0%
2. Education on change of diet	6	60%	4	40%	0	0%
3. Patient preferences	8	80%	2	20%	0	0%
4. Recommendations for diets	1	10%	6	60%	3	30%
5. Present different nutritional strategies	6	60%	3	30%	1	10%
6. Approach to weight-reduction	7	70%	3	30%	0	0%
7. Encouragement of physical activity	9	90%	0	0%	1	10%
8. Safety of physical activity	2	20%	8	80%	0	0%
9. Education of benefits from phys. activity	4	40%	5	50%	1	10%
10. Setting relatable goals	7	70%	1	10%	2	20%
11. Encouraging weight stabilization	2	20%	6	60%	2	20%
12. Behavioral therapy interventions	5	50%	5	50%	0	0%
Mean	6.4		4.6		1.0	

After analyzing the overall outcomes for each item on the checklist, Table 3 displays the compliance of each app with the German obesity guideline. The ratio between “yes” (green), “no” (red), and “unclear” (transparent) answers is visible as a color profile. For the DiGA “Oviva Direkt” no item was answered negatively. “zanadio” did not meet item 4 (“Recommendations for diets”). Consequently, two items (“Recommendations for diets”, “Present different nutritional strategies”) at “Oviva Direkt” and one item (“Encouraging weight stabilization”) at “zanadio” were answered with “unclear“. In the remaining eight apps, there were always at least two items marked as “no”. For example, “Foodvisor—Ernährung und Diät” did not show “Safety of physical activity” (item 8) and “Setting relatable goals” (item 10). The mobile health application “Fizz Up: Training und Ernährung” showed the most “no”’s, with nine negations. In this case, only item 3 (“Patient preferences”) and item 7 (“Encouragement of physical activity”) were answered with “yes”, while “Setting relatable goals” (item 10) was classified as “unclear”. Three apps (“DWP Fitness—Diät & Sport”, “FIT-UP: Fitness & Ernährung”, “wikifit—Kalorienzähler”) showed six negations each, resulting in 50% of the items not being met.

**Table 3:** Compliance with the German obesity guideline of each app.

App Item	1	2	3	4	5	6	7	8	9	10	11	12
DWP Fitness—Diät & Sport												
Fabulous—Gewohnheitstracker												
FIT-UP: Fitness & Ernährung												
Fizz Up: Training und Ernährung												
Foodvisor—Ernährung und Diät												
GlücksFigur—Abnehmen & Diät												
wikifit—Kalorienzähler												
Workouts Zuhause—Training												
Oviva Direkt^a^												
zanadio^a^												

1–12 marks the checklist items showed in Table 2.

Colors representing the answer options yes, no or unclear.

^a^ Official “Digitale Gesundheitsanwendungen” (DiGA).

The quality of the mobile health applications, represented via MARS-G mean score, varied between 2.93 and 3.89 (see Table 4). The mean overall MARS-G score for all apps included in the rating was 3.39 (SD = 0.39). The median was 3.24. The first section A focusing on the engagement received an overall mean score of 3.14 (SD = 0.57), with the apps differing between a score of 2.4 and 4.0. The engagement score therefore was the lowest overall mean score. Section B, targeting the functionality, showed scores between 2.75 and 4.0 and resulted in an overall mean score of 3.5 (SD = 0.43). The highest overall mean score with 3.57 (SD = 0.52) was achieved in section C, targeting the aesthetics. The values of the apps varied between 2.67 and 4.33. The last section D, focusing on the information, reached an overall mean score of 3.35 (SD = 0.45), with scores from the apps varying between 2.6 and 4.0.

**Table 4:** MARS-G quality scores.

App	Overall mean	Section A	Section B	Section C	Section D
		“Engagement”	“Functionality”	“Aesthetics”	“Information quality”
DWP Fitness—Diät & Sport	3.15	2.6	3.5	3.33	3.17
Fabulous—Gewohnheitstracker	3.78	3.8	3.5	4.0	3.83
FIT-UP: Fitness & Ernährung	3.15	3.0	3.5	3.33	2.8
Fizz Up: Training und Ernährung	2.93	2.8	2.75	2.67	3.5
Foodvisor—Ernährung und Diät	3.82	3.8	3.75	4.33	3.4
GlücksFigur—Abnehmen & Diät	3.0	2.4	3.25	3.33	3.0
wikifit—Kalorienzähler	3.32	2.6	4.0	3.0	3.67
Workouts Zuhause—Training	3.07	3.0	3.0	3.67	2.6
Oviva Direkt^a^	3.89	4.0	4.0	4.0	3.57
zanadio^a^	3.85	3.4	4.0	4.0	4.0
Mean values	3.39	3.14	3.5	3.57	3.35
(SD)	(0.39)	(0.57)	(0.43)	(0.52)	(0.45)

^a^ Official “Digitale Gesundheitsanwendungen” (DiGA).

## 4 Discussion

The systematic search identified ten mobile health applications, while two of them (“Oviva Direkt” and “zanadio”) are DiGA for the treatment of obesity in Germany. Each app included content based on at least two of the following topics: nutritional therapy, diet programs, exercise therapy, or behavioral therapy.

All apps assessed met some recommendations based on the German obesity guideline, but the range between the apps in terms of fulfillment of these recommendations is quite wide, between two and eleven items. In most cases, it was possible to decide on whether the content was included or not. One exception was the item “Recommendations for diets”, which remained “unclear” in four apps. This was due to the fact that although diets were mentioned in these apps, no further explanations or recommendations were provided for the diets.

The items mostly fulfilled in the apps were items 1, 3, 7, and 10.

Item 1 and 7, focusing on “Individual nutritional recommendations” and “Encouragement of physical activity” reflect two of the essential components of holistic treatment of obesity (13). A recent qualitative study investigated the personal motivation for weight loss in people with obesity. The majority of participants expressed their intention to eat healthily or increase their physical activity, but eventually lacked motivation and self-regulation to success. The participants reported a need for motivation boosters, such as reminders, to continue with healthy habits (35). A review about mobile apps for weight management also concluded that one of the biggest challenges regarding the use of apps targeting the treatment of obesity is to increase the motivation of the participants, especially in populations with lower adherence (36). Because longer treatment duration leads to positive effects (37), the motivation to continue using the app might be essential for an effective treatment. According to the German guideline for the treatment of obesity, motivation is an important aspect of long-term weight loss and weight maintenance (13). Similar results were discovered in a randomized controlled trial examining a personalized web-based weight loss program. The trial has shown that adherence is a key to success. Each additional session accomplished by participants led to an increased chance of a significant weight loss after 24 weeks by 2% (38).

Item 3, “Patient preferences”, requires the treatment to be patient-centered and consider individual preferences (13). Individual preferences were carried out differently in the apps. In most of the apps, it was possible to choose between different nutritional strategies and to exclude individual foods. Furthermore, fitness content most often showed various exercises from whom to choose and allowed patients to choose a workout-plan, which would most likely suit them. A systematic review and meta-analysis of personalized eHealth interventions for obese and overweight adults found that a combination of personalized content and customized human feedback can lead to the greatest treatment effects. The intervention may have a positive effect if personalization elements such as reminders, self-monitoring, and goal setting are included. The meta-analysis also showed a personalized digital treatment strategy significantly reduced participants' weight (39). Another systematic review of tailored eHealth interventions for weight reduction concluded that personalized content in eHealth weight loss programs is perceived by patients as more relevant, helpful, and understandable and at the same time may have a small effect on weight reduction (40). A study examining a personalized web-based weight loss program concluded personalized feedback given by a human led to a longer use of the program and a higher engagement rate, which could be especially beneficial for participants who would otherwise abandon the program quickly (38). The inclusion of personalized elements in mobile health applications may improve adherence and motivation, potentially enhancing the effectiveness of the treatment.

Item 10 “Setting relatable goals” was fulfilled by seven apps. The objectives ranged from a fixed date on which the weight was to be achieved, to weekly weight targets and individual motivational targets relating to specific life events. In a recent study examining the association between goal setting and weight reduction in a community weight loss program, participants were more willing to continue the program and lose weight if they set themselves reachable goals. Findings also revealed a medium weight loss goal had better effects than goals which were set below 10% weight loss (41). It can be assumed that it is important for a successful app to assist patients in setting manageable goals based on their individual measures to avoid demotivation.

While item 5 “Behavioral therapy interventions” was only fulfilled by 50% of the assessed apps, literature highlights these behavioral components can be the key element of any obesity treatment, both in face-to-face treatment and in digital solutions, leading to an overall increased effectiveness of the apps (36). A systematic review targeting cognitive behavioral therapy for obesity, concluded that behavioral therapy is an effective treatment of obesity leading to weight reduction. In combination with other methods, such as physical activity the treatment of obesity can become multi-dimensional and most effective (42).

Overall, it can be noted that using mobile health apps can achieve clinically relevant weight loss, help patients improve their self-regulation and therefore be overall effective (43). However, a systematic review and meta-analysis examining the effectiveness of smartphone apps for weight loss reported mobile apps achieved significant overall weight loss, but even greater when the mobile health app was used in combination with human behavior coaching or feedback. The highest weight loss was reached when the mobile health app was combined with a tracker and a behavioral intervention given by a human coach (44). This raises the question of whether blended care interventions, combining digital support with professional health coaching, might be an effective approach to treating obesity. Results of another recent systematic review on the effects of combined face-to-face and eHealth interventions for weight reduction showed how blended care approaches can lead to significant weight loss, an increase of physical activity and an improvement in quality regarding nutrition (45). Nevertheless, it is still a lack of research about what the blended care approach should look like in terms of the optimal design and frequency of personal contact.

The overall MARS-G mean score, assessing the quality of the apps, is 3.39 and therefore shows an overall acceptable quality. Compared to the other apps, the two DiGA have the highest MARS-G score. Both DiGA have proved their effectiveness in randomized controlled trials. “Oviva Direkt” demonstrated a significant weight reduction of 3.2% after 12 weeks and a weight maintenance after 24 weeks in the intervention group (46). The randomized controlled trial on the efficacy of “zanadio” showed an average significant weight loss of 7.75% in the intervention group within 12 months and a significantly improvement of the well-being, quality of life and waist to height ratio compared to the control group (47). In contrast, patients who can download the other mobile health apps free of charge cannot assess the effectiveness of these apps, because no evaluation was conducted. Nevertheless, especially section C regarding the aesthetics, showed the highest mean score. The section on engagement received the lowest mean score. Based on the already named need for personalized structures within the app to increase the motivation and adherence of the participants, the features leading to engagement are among the most important ones.

Trying to assess the acceptance of patients using the included mobile health apps, an important consideration is the access to these treatment options. The access for the eight assessed apps, which are no DiGA, can be described as low-threshold. This can be assumed, as the apps can be downloaded and used free of charge. Hence, these are treatment options for patients to test and use self-determined and without being prescribed by healthcare professionals. However, the DiGA are also free of charge if the patient consults a healthcare professional (physician or psychotherapist), and the DiGA is reimbursed by the SHI. Maybe the threshold is higher here because the patient first needs to contact the physician or the SHI to have a free access to these DiGA. According to a survey of insured and routine data from one German SHI, the majority of DiGA users became aware of this option because their physicians recommended it, and other information channels were rarely used (48). Although access may be more cumbersome, the DiGA offer a high quality with evidence for the content displayed, whereas the freely accessible mobile health apps do not provide evidence, making it difficult to assess quality from the patient's perspective.

The number of patients using DiGA increased over the last few years, leading to 374 thousand prescribed DiGA since 2020 (49). If the number of prescribed DiGA is compared to the total number of insured adults in the German SHI, which is around 61 million (50), it becomes apparent that a rather small proportion of the insured people utilize DiGA up to now. The survey conducted by a German SHI yielded similar results, with only 0.29% of all insured individuals taking advantage of digital health applications. These numbers suggest DiGA are not yet fully implemented in the treatment of diseases in Germany (48).

An online survey study analyzing the acceptance of mHealth apps in Germany in 2022 discovered 76% of the participants would use mHealth apps or DiGA. A younger age and higher digital competences (self-assessed) were indicators for the intention to use. For 53% of participants, a governmental quality control would be a prerequisite for the use of health apps. Additionally, for 67% of participants, it does not matter whether a health app is prescribed by a physician, which indicates they would be willing to use non-prescribed, freely available health apps. In total, only 27% of those surveyed indicated they would be willing to pay for a mobile health application on their own. Comparing the acceptance of mHealth apps and DiGA, 31% of the participants would only consider DiGA as an effective treatment addition, while 53% of the participants viewed health apps in general as a positive addition to the therapy. DiGA are mainly distinguished from other health apps by acceptance of the physician, medical performance, and data security. The study showed an overall high level of acceptance for mHealth interventions in Germany (51).

While the acceptance may be rather high from the patient perspective, it is just as relevant to perceive the acceptance of the healthcare professionals to increase the use of DiGA. A representative, national survey of healthcare professionals in 2022 explored the insights of DiGA in day-to-day care. A third of the respondents already gained experience in testing or prescribing a DiGA, but just 6.3% of them assigned DiGA more than fifteen times. 49.2% of the surveyed physicians would not prescribe a DiGA and even 77.8% of the respondents recognized obstacles in the deployment of DiGA. These included doubt about the effectiveness, concerns about the data safety as well as the excessive price. The survey demonstrated an increase in acceptance among healthcare professionals in 2022 compared to 2021, but about half of the respondents were not fully convinced about the benefits of the DiGA. The survey also pointed out how often healthcare professionals criticized the relation between DiGA costs and the reimbursement of their own services. Particular attention is given to the difference between a higher cost expenditure for the digital application and lower reimbursement for the medical service associated with additional time resources required to prescribe, explain and educate the patient (52). A recent qualitative study, where 38 GPs in Germany were asked about their experience with DiGA, also indicated a need for high-quality information and comprehensive training programs on DiGA. They would also like the SHI to educate patients about DiGA and to promote them to ensure they are not left alone with this task (53).

### 4.1 Limitations

A few limitations should be pointed out in this work.

First, this review was based on the methodology for systematic reviews according to the PRISMA statement (34). Nevertheless, the assessment of apps differs significantly from the assessment of scientific literature. Because of this, the search terms used in the assessment were single terms, trying to display what affected persons would search, including the disease itself but also highly associated terms, mostly applied in the linguistic usage. The possibility of an important search term not being used, is nevertheless existing. In addition, the previously defined aspect that the apps included must be available in both the Apple App Store and the Google Play Store can also be seen as a restriction.

Secondly, the systematic search only includes apps that are free of charge for patients: on the one hand, eight apps that can be downloaded free of charge from the App Store and, on the other hand, two DiGA. Additional apps that have to be paid for by the patients themselves (one-off or subscription) may also be available in the app stores. Literature suggests a correlation between higher app quality and higher prices (54, 55). For instance, the two DiGA, which cost the SHI € 220.90 (“Oviva Direkt”) and € 218.00 (“zanadio”) for three months each (26), already show a higher rating in the MARS-G score than the other eight available apps from the App Store. Perhaps some mobile health apps not listed as DiGA and only available behind a paywall are of better quality than free downloadable apps. However, it is still difficult for patients to assess the quality of these applications.

And third, the checklist used in analogy to the guideline is not a validated instrument. In addition, no criteria were formulated for determining when a recommendation was or was not fulfilled by an app. Moreover, no weighting was given to the points whose fulfilment is particularly important. In this context, it is important to mention that the German guideline for the treatment of obesity based on the last update from 2014 (13) may be outdated. Therefore, it should be considered here a change in the requirements for a successful treatment of obesity. As a result, the content displayed in the apps must be adapted. This can also be surmised with reference to the DMP for obesity, in which aspects such as sleep, psychosocial factors etc. are already addressed (19). Nevertheless, the problem of obesity not being a mental and behavioral disorder according to the ICD classification still exists, making accompanying psychotherapeutic treatment neither justified nor financed by SHI.

## 5 Conclusion

All mobile health applications assessed in this work, included at least two of the recommended topics, such as nutritional therapy, diet programs, exercise therapy, or behavioral therapy. In addition, all of them met some recommendations for the treatment of obesity from the German obesity guideline but varied rather widely in the number of recommendations met. The two DiGA included in this assessment fulfilled most of the recommendations and therefore stood out both in terms of content and the quality as assessed by MARS-G.

In principle, acceptance of mHealth seems to be quite high among the German population, whereas healthcare professionals still have concerns regarding safety and effectiveness aspects. To increase the effectiveness of mobile health applications, a personalized approach considering participants' preferences as well as engaging structures enhancing adherence and motivation are important. Overall, the use of an app may be an effective approach with an increased access to care for some obese patients. The use of a blended care treatment, for example the combination of the DMP and a mobile health app, may increase the effectiveness for patients with low adherence. Further research to find out if different patient groups may prefer and benefit from various treatment options for obesity, such as mobile apps, blended care, personal face-to-face contact or group settings, is necessary. In the future, it should also be researched if mobile applications and DiGA can reduce barriers and simplify the access to a holistic obesity treatment.
